# Are Female-Breadwinner Couples Always Less Stable? Evidence from French Administrative Data

**DOI:** 10.1007/s10680-024-09705-7

**Published:** 2024-06-13

**Authors:** Giulia Ferrari, Anne Solaz, Agnese Vitali

**Affiliations:** 1 Institut National d’Etudes Démographiques, Aubervilliers, France; 2 University of Trento, Trento, Italy

**Keywords:** Union dissolution, Divorce, Female-breadwinner couples, Income, Cohabitation

## Abstract

The paper studies the association between partners' relative incomes and union dissolution among couples in France. With the increase in dual-earner couples and women’s educational level, couples in which women earn more than their partners are structurally becoming more widespread. Because female breadwinning challenges long-lived social norms regarding traditional gender roles, scholars have theorized a higher risk of union dissolution among female-breadwinner couples compared to couples in other income arrangements. We estimate the risk of union dissolution using regression analyses on unique longitudinal data from French administrative sources containing an unconventionally high number of couples (4% of the population) and separation events (more than 100,000), as well as precise and reliable income measurement. Female-breadwinner couples face a higher risk of union dissolution compared to other couple types. This result is robust to various definitions of female breadwinning and controls for partners’ employment status. Contrary to recent research on other countries, we find no sign of a fading effect among younger cohorts. However, among younger, cohabiting couples and couples in registered partnerships the risk of union dissolution is lowest when both partners are employed and provide a similar share of the total couple’s income, suggesting the emergence of a new profile of stable couples. The female-breadwinner penalty in union dissolution is in place; also in France, it holds among married and cohabiting couples and registered partnerships, across all birth cohorts and levels of household income.

**Supplementary Information:**

The online version contains supplementary material available at 10.1007/s10680-024-09705-7.

## Background

Two unprecedented changes have transformed gendered patterns of employment and breadwinning within couples in recent decades. First, dual-earner couples have become widespread with the massive entry of women into the labor market. Second, the share of couples with women being more educated than their partners has increased in the developed world (Bouchet-Valat, 2018; Klesment & Van Bavel, 2017). These two trends imply that female-breadwinner couples, defined in this paper as couples in which the woman earns more than her partner, are becoming structurally more widespread (Esteve et al., 2016).

Female breadwinning challenges long-lived social norms regarding conservative gender roles. Therefore, scholars have theorized that female-breadwinner couples would be more exposed to the risk of union dissolution than other couple types. Empirical evidence supports such claims for the USA (Bertrand et al., 2015; Brines & Joyner, 1999; Killewald, 2016; Teachman, 2010), Australia (Foster & Stratton, 2021), and selected European countries (Jalovaara, 2003, 2013; Kalmijn et al., 2007). However, recent studies have reported interesting fading patterns. First, the female-breadwinner penalty is weak among recent American marriage cohorts (Schwartz & Gonalons-Pons, 2016), suggesting a decreasing penalty in union dissolution over time for female-breadwinner couples. One possible mechanism is that, as female breadwinning becomes more widespread, the risk of union dissolution among female-breadwinner couples becomes similar to the risk faced by other couple types. Second, some studies found no evidence of a female-breadwinner penalty in union dissolution among cohabiting couples (Ishizuka, 2018). Previous studies have also shown that the female-breadwinner penalty in union dissolution is context dependent. The risk of union dissolution is highest for female-breadwinner couples in countries and regions where gender equality is not supported by institutional arrangements (Cooke, 2006; Theunis et al., 2018) and where the support for the male-breadwinner norm is high (Gonalons-Pons & Gangl, 2021).

In France, as elsewhere, female breadwinning represents a non-negligible income arrangement. As of 2017, women earned more than their partners in one in four couples in prime earning age (author’s calculation from EDP data, see below), up from one in five in 2002 (Morin 2014). France is an interesting case study. The increase in non-marital cohabitation has been massive in France since the 1970s. Cohabiters now have long-lasting partnerships and are often parents: 61% of children are born outside marriage in 2021 (Insee, 2021). However, non-marital cohabiters still differ from married spouses, the former displaying more egalitarian values and behaviors. For example, unmarried couples are more egalitarian in housework sharing than married couples in several European countries, including France (Domínguez-Folgueras, 2013). This is also the case for couples in registered partnerships, in France referred to as PACS (“*pacte civil de solidarité”*). Introduced by the French Civil Code Act in 1999, PACS, established by a private or notarial act, provides legal recognition and tax advantages, although it offers less protection in matters related to inheritance and survivors’ pensions (in cases of a partner’s death) compared to marriage. PACS couples generally adopt more egalitarian behaviors, more often live in urban areas (Kandil & Périvier, 2021), and have a weaker attachment to gender roles than married couples (Rault, 2010). In terms of SES characteristics, married spouses and cohabiters have similar levels of education, whereas both male and female partners in a PACS are more highly educated (Kandil & Périvier, 2021). Married women are less likely to work and have more children than women in other partnership types. For comparison with the international literature, we will refer to PACS couples as registered partnerships in the remainder of the article.

The existence of these three partnership types (marriage, cohabitation, and registered partnership) makes France a fascinating setting for testing the association between female-breadwinning and union stability. Furthermore, female employment and dual earning in France have been common, socially accepted, and publicly supported for decades. In contrast with the USA, the French policy system supports gender equality and mothers’ employment with available, highly subsidized (thus affordable) childcare. While French mothers interrupt their careers after motherhood less often than in other European countries such as Italy or Germany, and they are now more educated than men on average, the gender pay gap is still sizeable (in the private sector) and motherhood penalties remain strong (Meurs & Pora, 2019). Paid parental leave—albeit not as generous as in the Nordic countries—and family allowances are in place, reducing the need to alter partners’ working hours and earnings after childbearing (Doucet, 2016). Therefore, we would expect female breadwinning to be more “acceptable” in France than in other countries where female employment is less socially and publicly supported. Although female breadwinning is different from dual earning, empirical results indicate that the tolerance toward female breadwinning may be associated with female employment rate and with prevailing gender norms. For example, Kowalewska and Vitali (2023) showed that, despite men and women in female-breadwinner couples tend to have lower levels of life satisfaction compared to any other couple types, the wellbeing penalty associated with female breadwinning is stronger in conservative contexts, such as in Germany compared to France.

This study investigates whether female-breadwinner couples are more likely to dissolve their union than other couple types. To the best of our knowledge, this is the first study to examine whether the female-breadwinner penalty in union dissolution also holds in France. Most existing longitudinal studies on the association between couples’ income (or working) arrangements and union dissolution are based on the USA (see, e.g., Killewald, 2016; Schwartz & Gonalons-Pons, 2016; Schwartz & Han, 2014; Teachman, 2010). A few exceptions include Kalmijn et al. (2007) for the Netherlands, Cooke (2006) for Germany, Jalovaara (2003, 2013) for Finland, Hamplová et al. (2021) for Canada, and Gonalons-Pons and Gangl (2021) for an international comparison between 28 European countries and the USA.

The originality of our study is threefold. First, the large sample size supports fine gradation of the relative income scale. This precision allows us to consider how the risk of union dissolution varies across the entire distribution of partners’ relative incomes, going beyond the two (female-breadwinner versus all other couples) or three-group comparisons (male breadwinners, equal earners, and female breadwinners) often used in the literature. Furthermore, because our measures of earnings and incomes are based on fiscal records, similar to Jalovaara (2003, 2013) for Finland and Kalmijn et al. (2007) for the Netherlands, our data are immune to self-reporting bias typical of survey data where, for example, men tend to overreport and women to underreport their incomes (Zagorsky, 2003). This bias is crucial when studying female breadwinning because partners may lie about their actual earnings so that they are not perceived as deviating from conservative male breadwinning norms (Atkinson & Boles, 1984). For example, men who reported earning about the same as their partners were shown to earn significantly less (Deutsch et al., 2003).

Second, previous studies found that the female-breadwinner penalty in union dissolution is common to both married and cohabiting couples, but with some heterogeneity in the comparison groups used: male breadwinning is more stabilizing for married couples, and equal earnings are more stabilizing for cohabiting couples (Brines & Joyner, 1999; Foster & Stratton, 2021; Kalmijn et al., 2007). Beyond comparing the risk of union dissolution across the distribution of partners’ relative incomes, this study further distinguishes between three types of couples: marriages, (informal) non-marital cohabitations, and registered partnerships. Partners in registered partnerships are on average more educated than partners in any other couple type and, similar to those in cohabiting unions, have more egalitarian attitudes than married spouses. Hence, we expect any deviation from equality among partners in registered partnerships (and, in particular, female breadwinning) to be associated with higher union dissolution, as found for cohabiting couples by previous literature based on other countries. However, registered partnerships are more similar to married couples in terms of their commitment to the relationship, legal protection, and income taxation, factors that do not necessarily favor dual earning and equality of incomes among partners as stabilizing for the couple.

Finally, we study a diversity of ages, ranging from 20 to 80 + years, highlighting unexplored age-cohort effects on the risk of union dissolution of female-breadwinner couples. In contrast with educational level, which is usually stable over time, couples’ income arrangements are not fixed across the partners’ life courses. Female breadwinning is short-lived (Winkler et al., 2005). Furthermore, virtually all previous studies have investigated the link between partners’ relative incomes and union dissolution focusing on active ages. However, union stability at later ages is worth studying because “gray” divorces are becoming increasingly common (Brown & Lin, 2012). Additionally, retirement can be a ‘turning point’ in late life and is associated with an increased risk of union dissolution (Bair, 2007), above and beyond changes in partners’ relative employment and incomes. Therefore, in this study, we broaden the age group of respondents used in previous studies. By considering older ages, we shed light on whether the association between relative incomes and union dissolution, which was found in previous research, holds across the life course or is peculiar to certain ages and life stages.

## Background and Research Questions

### Female Breadwinning and Union Dissolution

Specialization theories consider a gendered division of roles to be functional and optimal; relatedly, they attribute the rise in divorce observed from the second half of the twentieth century to the deviation from gender role specialization that coincided with women’s entrance into the labor force (Bales & Parsons, 1955; Becker, 1991; Parsons, 1949). The independence hypothesis sees the deviation from a gendered division of roles as challenging patriarchal gender norms and has stressed the negative effect of women’s incomes on union stability, as access to economic resources empowered women to leave unhappy partnerships (Oppenheimer, 1997; Sayer & Bianchi, 2000). These theories are somewhat outdated, as women’s employment is much more common, widely accepted, and frequently necessary for families in response to macroeconomic changes that have transformed men’s employment characteristics (Esping-Andersen, 2002; Ruggles, 2015). Also, empirical evidence has confirmed that women’s earning capacity has no effect on the probability of divorce at the individual level (Burgess et al., 2003).

However, while women’s contribution to the family’s incomes is widely accepted, and equal-earner couples have become widespread, female-breadwinner couples, i.e., couples where the woman out-earns the man, regardless of whether she is the sole wage earner or not, are still relatively unusual. From a cultural perspective, female breadwinning represents a deviation from a long-lived norm where the man is the main provider of resources for the family, and the woman is the caregiver for the home and children. Couples may still linger toward conservative gender roles. Such gender identity norms could be responsible for the higher risk of union dissolution experienced by female-breadwinner couples than couples in other income arrangements (Bertrand et al., 2015; Gonalons-Pons & Gangl, 2021).

Female breadwinning is also linked to men’s poor economic characteristics (Kowalewska & Vitali, 2021; Vitali & Arpino, 2016), which is also associated with couple instability. Killewald (2016) found that married American couples are more likely to divorce when husbands are not employed full time. Full-time employment and contribution to (at least some) income remain two important elements of men’s identities (Doucet 2016; Rao, 2020). By reversing symbolic gendered roles and undermining the fundamental traits of hegemonic masculinities, female breadwinning challenges couple stability in many ways.

More generally, female breadwinning is linked to economic difficulty, as female-breadwinner couples tend to have the lowest incomes of all couple types (Kowalewska & Vitali, 2021; Winslow-Bowe, 2006). In turn, financial strain and economic uncertainty may put the relationship under strain and be associated with union dissolution (Jalovaara, 2003; Ono, 1998). Hence, the role of absolute economic resources is crucial for understanding the true association between partners’ relative incomes and risk of union dissolution (Oppenheimer, 1997).

The female-breadwinner penalty in union dissolution was especially strong when women’s employment and economic resources were limited and male economic dominance was normative (Bertrand et al., 2015; Killewald, 2016; Sayer & Bianchi, 2000). With the reversal of the gender gap in education, couples where women are more educated than their partners become widespread, and female breadwinning may too become structurally more widespread (Esteve et al., 2016). As men are increasingly seeking traits associated with economic success in their prospective partners (Blossfeld, 2009), female breadwinning may become more socially accepted as well. As women’s roles as workers and income providers become more widespread and socially accepted, the risk of union dissolution for female-breadwinner couples may decrease. In support of this hypothesis, some studies have found that the female-breadwinner penalty is weaker among recent marriage cohorts in the USA (Schwartz & Gonalons-Pons, 2016), especially among the higher educated (Stratton 2021; Swartz & Han, 2014). Other studies have found that the female-breadwinner penalty in union dissolution is lower in contexts characterized by higher gender equality (Cooke, 2006; Lippmann et al., 2020), higher prevalence of non-traditional couples (Theunis et al., 2018), and weaker support for the male-breadwinner norm (Gonalons-Pons & Gangl, 2021). However, this evidence is mixed. Foster and Stratton (2021) found that the female-breadwinner penalty in union dissolution is higher—not lower—among younger couples. For Canada, Hamplová et al. (2021) found no evidence of a reduction in the female-breadwinner penalty in union dissolution during the past 30 years. Jalovaara (2003, 2013) found evidence of a female-breadwinner penalty in Finland, a country whose institutional characteristics would lead us to imagine the absence of such a penalty. In the international comparison, France is similar to the Finnish context in terms of female employment and public child care facilities. Hence, our study tests whether the Finnish “paradox” found by Jalovaara (2003, 2013) is also observable in France.

Our first research question is: Are female-breadwinner couples more likely than couples in other relative income arrangements to dissolve their union in France, a country where dual earning has been common and socially supported for decades?

### Marriage, Cohabitation, and Registered Partnerships

Although cohabiting couples are becoming more widespread across the developed world, the association between the risk of union dissolution and partners’ relative incomes has mainly been studied for married couples. Studying union dissolution among non-married couples is particularly relevant in the French context because marriages, non-marital cohabitations, and registered partnerships (PACS) co-exist in France.

Hence, our second research question is: Does the risk of union dissolution for female-breadwinner couples differ across union types?

Among married couples, many studies (Bertrand et al., 2015; Foster & Stratton, 2021; Kalmijn et al., 2007; Killewald, 2016; Schwartz & Gonalons-Pons, 2016) find that the risk of divorce increases with the woman’s share of couple income or when the husband is not employed. Although male-breadwinner married couples are generally more stable, empirical results for dual-earner couples are not consistent. Some studies found that equality of incomes destabilized marriage in the USA (Rogers, 2004) and the Netherlands for short union durations (Kalmijn et al., 2007). Others found equality of incomes to be stabilizing, for example, in Canada (Hamplová et al., 2021), Finland (Jalovaara, 2013), and, for long union durations, also in the Netherlands (Kalmijn et al., 2007).

From an institutional point of view, marriage is the most regulated union, followed by registered partnerships, then cohabitation, which has little legal protection, obligations, and tax rules—see Kandil and Perivier (2021) for a comparison of the three types of marital statuses. Cohabiting couples generally have a higher risk of union dissolution than married couples (Liefbroer & Dourleijn, 2006; Lyngstad & Jalovaara, 2010; for a review on how union dissolution risk changes across married and cohabiting couples see Jalovaara, 2013). Cohabiting unions lack the legal recognition granted by the marital contract; hence, it is easier and less costly to exit from a cohabitation than a marriage (Brines & Joyner, 1999).

Furthermore, partners in cohabiting unions and even more in registered partnership (Kandil & Perivier, 2021) have higher gender-egalitarian attitudes (Kaufman, 2000) and behaviors compared to married spouses (Domínguez-Folgueras, 2013), for instance, in terms of their division of paid and unpaid work. Hence, equality of incomes among partners would be more stabilizing for cohabiting unions and registered partnerships than for married ones. Any deviation from a situation of equality would increase the risk of union dissolution, particularly when the deviation is toward female breadwinning (Brines & Joyner, 1999). However, the violation of conservative norms associated with female breadwinning may be smaller for cohabiting couples than for married couples due to cohabiters’ more progressive beliefs. Especially in contexts characterized by a large diffusion of non-marital cohabitation, such as France, married individuals are likely to be highly selected in terms of conservative ideals and support for male breadwinning (Liefbroer & Dourleijn, 2006, Kandil & Perivier 2021); hence, the female-breadwinner penalty in union dissolution may be stronger among married couples (Gonalons-Pons & Gangl, 2021).

Studies that distinguished between married and cohabiting couples found heterogeneous results regarding the existence of a female-breadwinner penalty in union dissolution among cohabiting couples. Brines and Joyner (1999), Jalovaara (2013), and Kalmijn et al. (2007) agree that for both married and cohabiting couples, the risk of union dissolution is higher for female-breadwinner couples than for couples with other income arrangements. Foster and Stratton (2021) found that the female-breadwinner penalty holds, especially among young cohabiters in the USA and Australia. In contrast, Ishizuka (2018) found no female-breadwinner penalty among cohabiters in the USA. In addition, there is no consensus on which income arrangements are more stabilizing for cohabiting and married couples. Brines and Joyner (1999) and Kanji and Schober (2014) find that equality of incomes among partners is associated with higher union stability among cohabiting couples in the USA and UK, respectively. Hamplová et al. (2021) found that income equality stabilizes both cohabiting and married couples in Canada, whereas Jalovaara (2013) did not find this stabilizing effect for cohabiting couples in Finland. Kalmijn et al. (2007) found that equality of incomes is stabilizing only for cohabiting couples who have been together for less than five years in the Netherlands. However, for longer union durations, the risk of union dissolution increases with the woman’s share of total income for cohabiting and married couples alike. For the USA and Australia, Foster and Stratton (2021) found that the female-breadwinner penalty is especially strong among cohabiting couples, whereas Jalovaara (2013) found the opposite for Finland.

Compared to non-marital cohabitations, registered partnerships are more committed unions, similar to the Nordic countries (Wiik et al., 2009). The union dissolution costs are lower for registered partnerships than for married couples but generally higher than for cohabiting couples. Hence, we expect the risk of union dissolution across all constellations of relative incomes for registered partnerships to mimic that of married couples. Also, registered partnerships in France, like married couples, benefit from legal protection in the case of union dissolution and (since 2022) benefit from joint taxation rules that favor unequal incomes, whereas joint taxation is not allowed for non-marital cohabitations. Thus, because the institutions of marriages and registered partnerships protect and incentivize unequal earning, we expect the risk of union dissolution among single-breadwinning couples (both male and female) to be highest among non-marital cohabitations.

Registered partnerships, however, also share similarities with cohabiting couples: partners in both registered partnerships and cohabiting couples retreat from marriage and are more likely to report favoring gender equality (Rault & Letrait, 2015) compared to married partners. Hence, since married couples hold more traditional norms and values compared to non-married couples, we expect equality of incomes to be more stabilizing for cohabiting couples and registered partnerships, compared to married couples and we expect the association between female breadwinning and union dissolution to be strongest among married couples.

### A dynamic Approach to Female Breadwinning

Partners’ relative incomes are not static; they evolve in response to major life events such as transitioning from school to work, childbearing, unemployment, retirement, or illness. Winslow‐Bowe (2006) and Kanji and Schober (2014) explicitly called for a life course approach to best understand the meaning and consequences of female breadwinning for couples’ outcomes. First, a dynamic approach is useful because female breadwinners are frequently short-lived. Winkler et al. (2005) showed that, among American married couples at the end of the 1990s, the female-breadwinner arrangement lasted less than three consecutive years for 40% of couples. Additionally, Winslow‐Bowe (2006) showed that a minority of women out-earned their husbands for five consecutive years, using American data for a cohort of married individuals born in 1979. Kanji and Schober (2014) found that the equal or main earning status of mothers of young children in Britain lasts only a few years.

A dynamic approach is also useful because partners’ relative incomes depend on certain life stages and the female breadwinner status may have different meanings across the life course.

Hence, our third research question is: Does the relationship between female breadwinning and union dissolution change across ages?

At younger ages, upon entering into the labor force, gender pay gaps are low, and partners’ relative incomes tend to be more similar. Hence, young women might be more likely to out-earn their partners than women in prime earning age (Winslow‐Bowe 2006). Throughout their life course, women’s incomes might grow more slowly than men’s because the couple might prioritize men’s over women’s careers, or because of gender/motherhood-wage discrimination in the labor market, thereby reducing women’s likelihood of out-earning their partners. Later, older women might be more likely to out-earn their partners, because they retire later their partner since they are on average younger (on average 2.4 years in our sample). Winslow‐Bowe (2006) found that childbirth, age of children, age, and partnership duration are all important in determining year-on-year changes in partners’ relative incomes. In addition, partners’ relative incomes may change over a couple’s life course due to partners taking turns in breadwinning and caring roles (Gerson, 2010) or in response to external factors such as unemployment, end of a short-term contract, or promotion.

Lastly, as previously mentioned, the empirical evidence is mixed about whether the female breadwinner penalty has reduced over time. Schwartz & Gonalons-Pons, 2016 found a weaker female-breadwinner penalty in union dissolution among recent marriages in the USA, whereas Foster and Stratton (2021) found a positive penalty among younger couples. For Canada, Hamplová et al. (2021) found no evidence of a reduction in the female-breadwinner penalty across three decades. Although not directly comparable due to their different sample choices, they suggest that the relationship between relative income and dissolution risk may be changing over time.

Here, we adopt a dynamic approach that considers entry into and exit from the female-breadwinning arrangement. Following Winslow‐Bowe (2006), we also consider the presence and age of children, their own and partner’s age, and partnership duration (limited to married couples and registered partnerships due to data limitations) as important life stages. Finally, in order to measure possible fading or reinforcing effects of the female-breadwinner penalty among younger generations, and because relative incomes may have different meanings according to the life stages, we stratified our sample by ten-year age classes. Unlike previous studies that mainly considered active ages, we extend the observation window to also include retirement ages. This is particularly important in the context of increasing “gray divorce” that has been observed in the USA (Brown & Lin, 2012) and Europe recently (Solaz, 2021). This is also important because the retirement of one partner affects the relative resources of the couple and may temporarily change the couple’s breadwinning status.

### Female Breadwinners vs. Women as Primary Earners

So far, we have used the term “female breadwinners” to denote couples with women earning more than their male partners. However, this broad definition of female breadwinners encompasses two essentially different couple types: dual-earner couples with women out-earning their partners and couples where women are the sole wage earners. Operationally, female breadwinners can be defined in two ways: comparing partners’ relative employment status or their relative incomes. An employment-based definition identifies female breadwinners as women who are employed with a non-employed partner, whatever the reason (inactive, unemployed, or retired)—we refer to this couple type as ‘sole’ female breadwinners. A second approach is to use a strictly monetary definition and compare the individual incomes of both partners. According to this second definition, female-breadwinner couples are dual-earning partnerships, with women having individual incomes higher than those of men. We refer to this couple type as women as primary earners.

The relative employment versus relative income definitions yield two couple types that differ substantially in terms of their economic characteristics and their drivers into such income arrangements: ‘sole’ female breadwinners tend to have low household incomes, lower than those of male breadwinners, and low levels of education, whereas women as primary earners tend to have higher household incomes, comparable to those of men as primary earners, and high levels of education (Kowalewska & Vitali, 2021).

Hence, our fourth and final research question is: Are ‘sole’ female-breadwinner couples where women are the sole earner more exposed to the risk of union dissolution than dual-earner couples where women out-earn their partners?

Empirical studies suggest that couples generally become ‘sole’ female breadwinners by constraint, following the man’s job loss, rather than according to a genuine choice (Chesley, 2011; Chesley & Flood, 2017; Vitali & Arpino, 2016). In other words, ‘sole’ female breadwinning is frequently the unplanned outcome of economic necessity linked to economic shocks, which may add stress to the relationship quality and the wellbeing of partners, further contributing to an increased risk of union dissolution.

It may be acceptable for partners to be in a dual-earning couple with a primary earner woman, especially those with higher education, who also hold greater gender-egalitarian attitudes (Chesley, 2011) and higher absolute incomes (Kowalewska & Vitali, 2021). In contrast, ‘sole’ female breadwinning challenges the most important trait of hegemonic masculinities and male identities: the ability for a man to work and provide at least some income for their families. Hence, we expect the risk of union dissolution among dual-earning couples with women as primary earners to be lower than in ‘sole’ female-breadwinner couples where men are not employed.

## Data, Methods, and Variables

We used a recently released administrative database, the French Permanent Demographic Sample, which links censuses, vital event registrations, housing, and income tax declarations for 4% of the French resident population, representative of the French population. The dataset enables to track information on individuals born on 16 calendar days each year, called EDP individual. These individuals are those born on the first four days of each quarter for quarters 2, 3, and 4, and from January 2nd to January 5th for quarter 1. We focused on the cohort of EDP individuals aged 18 and over who were in a co-resident partnership (either married, in a registered partnership or a cohabiting union) at the beginning of the observation window on January 1, 2011, and followed them until January 1, 2017.

We dropped records where one or both partners’ income was missing (about 6%) and kept couples observed for at least two subsequent years. The final dataset was composed of 992,217 couples and 5,536,503 couple-years at risk of dissolution. Dissolution by partner’s deaths is considered as right censored. They are only observed for married couples and a subset of registered partnerships. It is not possible to distinguish separations due to partner’s death for cohabiters. Fortunately, as partner’s death concerns overall old couples who are in most cases married, this limitation affects our results only marginally. For each year, we observe the possible union dissolution, income, and work transitions for all couples.

The yearly risk of union dissolution was modeled with discrete-time event history models through binary logistic regression. Standard errors were clustered at the individual level. Our dependent variable accounts for whether a co-residential partnership at time t dissolves in the following year t + 1. We identified this event with a change in the marital status declared in the tax returns for married and registered partnerships and a with change in household composition for cohabiting unions. In the observation window, 95,538 union dissolutions occurred (36,016 divorces, 5,343 dissolutions of registered partnerships, and 54,179 dissolutions of non-marital cohabitations). On average, approximately 1.4% of couples dissolved in our sample each year.

Our primary independent variable is the woman’s share of the couples’ total income. For each partner, income is defined as the sum over the year of all individual incomes (from wage, positive self-employment income, unemployment allowances, and retirement pension) declared in the fiscal return. During the year, individuals may receive income from different sources, for example, from unemployment allowance and wages or from retirement pension and wages. We build a discrete scale measuring the woman’s share of the couple’s total income. This scale includes situations close to ‘sole’ male breadwinners (i.e., the woman’s share is 0 to 5%) and ‘sole’ female breadwinner (i.e., the woman’s share is 95–100%), along with a range of intermediate situations. We used an eleven-item categorization (with 9 intermediate positions in 10% intervals ranging from (5–15%] to (85–95%]) for the analyses and a five-item categorization for descriptives (with three intermediate positions: (5–45%], (45–55%], and (55–95%]).

Beyond relative resources, absolute resources add to the risk of union dissolution. Hence, we control for the couple’s total income (in quintiles) and the homeownership status (owners, private renters, and social renters) as a proxy for wealth.

We then control for important markers of life stages such as partners’ average age in ten-year classes, the presence of children in the household, and age of the youngest child (0–3, 4–8, 9–12, 13–18, 19 years and above, unknown age), to control for the stabilizing effect of having young children (Harkonen, 2014).

Other control variables included demographic aspects, such as union type (i.e., marriage, registered partnership, or cohabitation), the age difference between partners (i.e., man’s age minus woman’s age), and whether one of the two partners was born abroad, because the risk of union dissolution may be higher among partners from different cultures (Milewski & Kulu, 2014). Finally, we controlled for the population density of the place of residence (< 2,000, 2,000–20,000, 20,000–2 million inhabitants, the Paris area, and missing information) as divorce rates has been found to be higher in urban than in rural context (Kulu, 2012).

All time-varying covariates were measured the year preceding union dissolution.

We did not include the partners’ level of education in our main specification because of the high percentage of missing information on this variable in our data. Education is not available for all years but recovered when individuals are identified by the census, which take place from 1 to 6 years before the observation. For EDP individuals, the information is recovered for 75% of the sample, but much less for partners since they may not be in a co-residential relationship at the time of the previous census. We decided not to impute the educational level (since missing values could be also linked to recent couple formation) but to introduce it only in the robustness check with a dummy indicator when missing. Our data allowed us to reconstruct the formal duration of the union (0–4, 5–9, and 10 + years) only for married couples or registered partnerships. Instead, for cohabiting couples, we did not have information on the union duration. For this reason, we did not include union duration in our main specification but performed some robustness checks.

To answer our research questions and because we expected the associations between female breadwinning and risk of union dissolution to be stratified by crucial variables, we also considered interactions between our main independent variable (i.e., couples’ income arrangements) and the following variables: men’s employment situation (employed with positive labor income, unemployed with positive benefits, retired, and inactive without any labor-related income), couples’ total income quintiles, marital status (and couple duration when possible), and age class.

## Results

### Socio-Economic Characteristics of Female-Breadwinner Couples

Table 1 presents descriptive statistics. Half of the couples in our sample (49.3%) have a man as the primary earner (i.e., woman contribute between 5 and 45% of the couple’s total income). Equal earners (i.e., women contribute between 45 and 55% of the couple’s total income) represented about one-fifth (20.5%) of our sample. Women are primary earners (i.e., contribute between 55 and 95% of the couple’s total income) in 13.7% of the couples.

**Table 1:** Couple’s characteristics by woman’s relative income share (Column %)

	Total	Male breadwinner [0%-5%]		Equal earner	Female breadwinner	
		Sole MBW [0–5%]	MPE (5–45%]	(45–55%]	WPE (55–95%]	Sole FBW (95–100%]
*Woman’s age class*						
20–29	6.07	5.29	5.48	8.10	6.12	5.39
30–39	19.72	19.07	18.50	22.48	20.60	20.21
40–49	22.60	22.12	21.83	22.97	24.12	30.95
50–59	21.63	27.48	18.89	19.97	26.03	33.67
60–69	18.13	17.81	19.35	17.08	17.14	8.04
70 +	11.85	8.24	15.96	9.41	5.98	1.74
Mean age difference among partners (M-W)	2.38	3.38	2.18	2.16	2.40	2.21
*Union type*						
Marriage	82.84	91.30	84.61	76.90	77.00	79.06
Pacs	5.00	2.25	4.84	6.76	5.98	4.18
Cohabitation	12.15	6.46	10.55	16.34	17.03	16.76
*Age of the youngest children*						
No children	44.84	40.42	47.29	44.06	43.21	35.39
0-3y	7.12	10.14	6.39	7.20	6.46	7.00
4-8y	14.53	15.62	13.81	15.14	14.74	16.46
9-12y	8.25	7.91	8.09	8.31	8.70	10.89
13-18y	10.29	10.24	10.01	10.21	10.95	14.01
19y +	6.98	7.80	6.66	6.53	7.71	8.67
Unknown age	7.99	7.86	7.75	8.54	8.23	7.58
*Population density*						
Rural	27.01	22.32	27.21	29.55	27.66	25.45
2-20 k	19.09	18.02	19.81	19.41	17.61	15.81
20-200 k	18.63	21.11	18.56	17.46	17.83	19.87
200-2 m	21.88	24.46	21.93	20.52	21.03	21.99
Paris area	13.38	14.06	12.48	13.05	15.86	16.85
Unknown density	0.01	0.03	0.01	0.01	0.01	0.03
*Couple's nativity*						
Both born abroad/natives	94.50	92.42	94.92	95.55	94.04	91.33
Mixed couples	5.50	7.58	5.08	4.45	5.96	8.67
*Home ownership status*						
Owner	77.07	66.54	79.91	79.42	76.86	60.89
Social renter	8.47	16.08	6.94	6.64	7.58	15.93
Private renter	14.45	17.39	13.15	13.94	15.56	23.18
*Man’s professional situation*						
Employed	61.41	63.28	61.04	66.57	60.12	11.94
Unemployed	3.38	5.16	1.73	2.77	8.58	1.76
Retired	33.54	31.52	37.22	30.64	31.19	2.91
No income	1.67	0.04	0.01	0.02	0.11	83.39
*Quintiles of total income*						
1st	18.07	47.08	13.74	7.61	11.46	67.68
2nd	20.19	20.28	22.68	16.17	17.85	15.80
3rd	20.68	10.09	20.38	28.68	23.00	6.58
4th	20.61	8.25	20.40	28.25	25.32	4.32
5th	20.45	14.30	22.80	19.30	22.37	5.62
Mean total income (€)	46,134	38,567	48,066	47,159	48,820	24,117
Total	100	14.51	49.29	20.50	13.74	1.97
Person-years	5,536,503	803,203	2,728,736	1,134,893	760,583	109,088
Union dissolution events	95,538	9,984	41,900	22,018	18,105	3,531

Only 2% of the couples in the sample were ‘sole’ female breadwinners (i.e., women contribute between 95 and 100% of the couple’s total incomes), against 14.5% of ‘sole’ male-breadwinner couples.

Dual-earner couples with women as primary earners are essentially different from ‘sole’ female breadwinners. Over 65% of ‘sole’ female breadwinners are found in the lowest income quintile, by far the highest percentage in the sample, against only 11% of women as primary earners. Men in ‘sole’ female-breadwinner couples essentially have no income (83%) or are employed with very little income in the reference period (12%). Men in couples with women as primary earners have positive income from employment (67%) or pension (31%). Only a minority of men were unemployed in both groups, with a positive income (e.g., from social benefits). ‘Sole’ female breadwinners have the lowest homeownership rate in the sample, equal to 61%, against 77% for women as primary earners. ‘Sole’ female-breadwinner couples were less often without children in the household than women as primary earners and women in other income arrangements. They also have the highest share of couples with a partner born abroad.

However, women as primary earners and ‘sole’ female breadwinners share some demographic characteristics. Both couple types are more frequent among women in their forties and fifties and less frequent among women aged 60 and over. Compared to couples in other income arrangements, they are also more likely to be in non-marital cohabitations or registered partnerships than married and are more prevalent in the Paris area.

### Female Breadwinning is Not a Permanent Income Arrangement

**Fig. 1:**
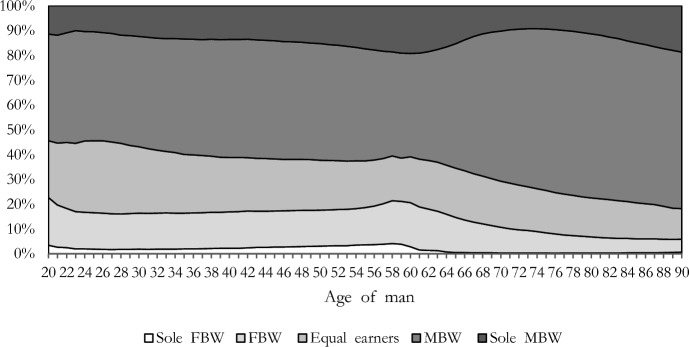
Types of couples by man’s age and relative income. *Note*: Sole FBW = the woman earns between (95–100%] of the couple’s total incomes; FBW = the woman earns between (55–95%] of the couple’s total incomes; Equal = the woman earns between (45–55%] of the couple’s total incomes; MBW = the woman earns between (5–45%] of the couple’s total incomes; Sole MBW = the woman earns between [0–5%] of the couple’s total incomes

Figure 1 shows how couples’ relative incomes change across the life course according to the man’s age. For all ages, the most widespread income arrangement is the couple with men as primary earners, followed by equal earners and couples with women as primary earners. The shares of ‘sole’ female-breadwinner and women-as-primary-earner couples are relatively stable during men’s working age. At the beginning of men’s working life and around retirement age, instead, female breadwinners and women as primary earners are relatively more frequent.

**Fig. 2:**
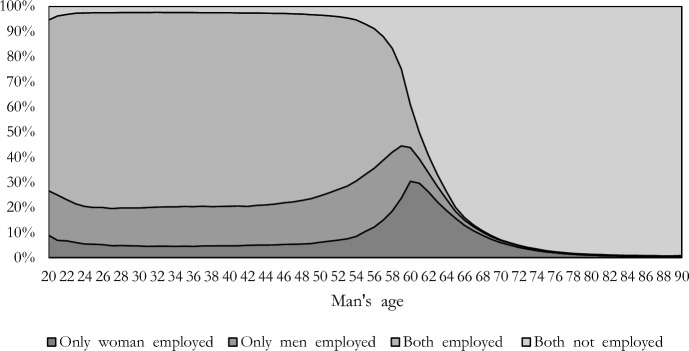
Types of couples by man’s age and relative employment

This is confirmed when we look at the partners’ relative employment status by the man’s age (Fig. 2). The share of couples with an employed woman and a non-employed man reaches 30% when the man’s age is around 60 years old. Among dual-earning couples, retirement generally occurs earlier for men than women because men have acquired more retirement rights than women (because of fewer career interruptions) and because they are on average older than their partners. This time gap could create a temporary situation in which the woman is still employed, and the man has already retired, which could lead women to temporarily out-earn men if the man’s pension is lower than the woman’s incomes—until the woman also retires. At later ages, when women retire, the share of couples with women as primary earners declines and the share of men as primary earners increases. These descriptive analyses clearly show that couples’ relative employment status and relative incomes are not static across the life cycle.

**Fig. 3:**
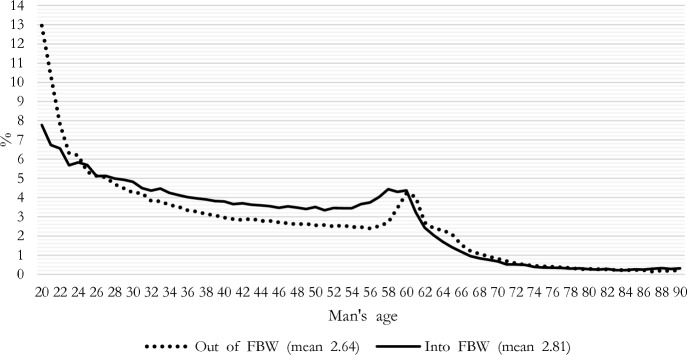
Percentage of couples moving into and out of female breadwinning by man’s age. *Note*: Indexes are computed in the following way: Out of FBW: Percentage of couples in which woman’s income in t-1 is below 55% of couple income in t-1 and woman’s income in t is above or equal to 55% of couple income in t. Into FBW: Percentage of couples in which woman’s income in t-1 is above or equal to 55% of couple income in t-1 and woman’s income in t is below 55% of couple income in t. *Reading note*: Among couples with a 40-year-old men, 3% are exiting the female-breadwinning status with respect to the previous year, whereas 3.8% are entering the female-breadwinning status

Another way to emphasize the dynamic nature of relative income is to look at the transitions from or into female breadwinning. Figure 3 considers the yearly change in the percentage of couples becoming female breadwinners and those who exit female breadwinning by the man’s age. Changes are more frequent at the beginning and end of men’s active ages. For instance, when men reach age 60, many retire—they exit dual earning—and, because retirement pension may be lower than the salary earned in the final years of employment, in some cases retired men may earn less than their still-employed female partner, particularly if they had similar incomes before man’s retirement. We first observe a clear peak in entries into female breadwinning because of male retirement and some years later a peak in exits corresponding to women’s retirement.

### The Risk of Union Dissolution and Partners’ Relative Incomes

**Fig. 4:**
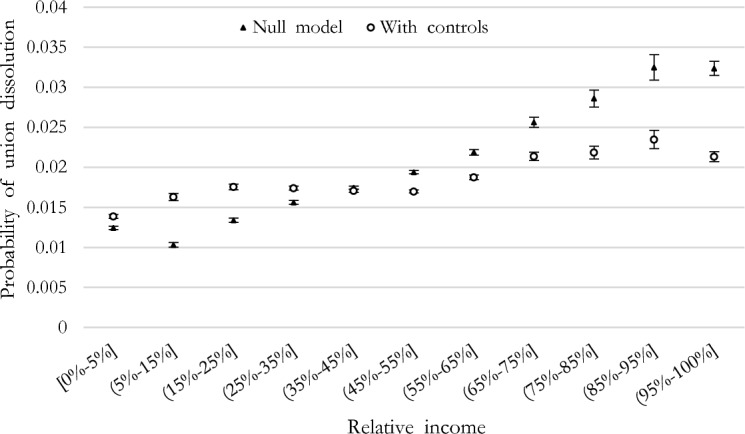
Predicted margins of union dissolution by woman’s relative income share (with 90% confidence intervals)

Results from logistic regression models estimating the risk of union dissolution by the woman’s share of couples’ incomes are presented in Table A1 in Appendix. To ease the interpretation of results, we report figures showing the predicted probabilities of union dissolution by key explanatory variables. Figure 4 presents the null model, suggesting that the larger the woman’s share of couple’s total income, the higher the predicted probability of union dissolution. The relationship seems almost linear and is confirmed by further descriptive statistics reported in Figure A2 in Appendix that uses a continuous version of the variable (100 levels) and is robust to a 5-item categorization (not shown). Exceptions are the two extreme categories: the lowest probability of union dissolution is not found for ‘sole’ male-breadwinner couples, but for couples where both partners are employed, with the woman earning a very small share of the couple’s total incomes 5–15%. At the other extreme, the probability of union dissolution for ‘sole’ female breadwinners is not significantly different than for dual-earner couples with women earning between 85 and 95% of the couple’s total income.

The inclusion of control variables—particularly the type of union—flattened the association between partners’ relative incomes and union dissolution (Fig. 4). The probability of union dissolution is now lowest for ‘sole’ male-breadwinner couples, followed by couples with women contributing between 5 and 15% of the couple’s total incomes. Couples with women contributing between 15 and 55% of the couple’s total incomes (hence, couples with men as primary earners and equal earners alike) face similar predicted probabilities of union dissolution. In other words, we observe a plateau for equal-earning couples. Instead, the probability of union dissolution significantly increases when women contribute more than 55% of the couple’s total income and continues to increase with the woman’s contribution to the couples’ income up to a share of 85–95%. The discontinuity observed at 55% of the woman’s share of couples’ incomes (from 53% of the total income when using the more detailed variable in Figure A2) suggests that women who earn just above 50% are similar to equal earners, whereas as the earnings difference increases, so does the risk of union dissolution. Thus, according to our first hypothesis, we find evidence of a female-breadwinner union dissolution penalty, increasing with the woman’s share of couple’s income as soon as the woman out-earns her partner. Once controls are included, the risk of dissolution remains 11% (exp(0.320)/exp(0.215)) and around 30% higher when the woman’s share of the couples’ total income is between 55 and 65% and 65–85%, respectively, relatively to equal-earner couples. However, net of absolute resources and other controls, for ‘sole’ female breadwinners, the predicted probability of union dissolution is significantly lower than for couples with women contributing between 85 and 95% of the couple’s total income.

**Fig. 5:**
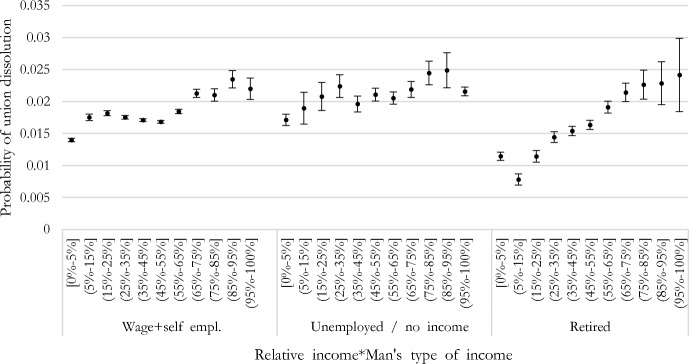
Predictive margins of union dissolution by woman’s relative income share and man’s type of income, logit models (with 90% confidence intervals). *Note*: For sample size issue, unemployed and no income categories have been grouped

In an additional model, we interacted the female income share of the couple’s income with the man’s employment status. Figure 5 shows the predicted probabilities of the interaction terms. Couples with a retired man are less likely to separate, on average, compared to couples with an employed or non-employed man (either unemployed or inactive, grouped for sample size issues). However, such differences are statistically significant only for couples where the woman’s share of the couple’s total income ranges between 0 and 5% and 35–45% (*p* < 0.001 for all differences contrasting couples with a retired vs. employed man and couples with a retired vs. unemployed/inactive man, results not shown). Instead, the risk of union dissolution is similar (i.e., not significantly different) for couples with women as the primary or sole income providers (i.e., earning between 45 and 55% and 95–100% of the couple’s total income), independently of whether the man is retired, unemployed, or inactive men (*p* > 0.05). The risk of union dissolution is significantly lower among equal-income couples (i.e., women provide 45–55% of the total income) with retired men than among equal-income couples with unemployed/inactive men (diff. = − 0,005, *p* < 0.001), whereas no significant difference exists for equal-income couples with retired vs. employed men (diff. = 0.000, *p* > 0.05). In other words, irrespective of the man’s employment status, couples with women as the primary or sole income providers are more likely to separate than other couple types. This means that a man’s employment status (unemployment/inactivity or retirement vs. employment) does not drive the female-breadwinner penalty. The increasing gradient is more pronounced for retired individuals.

**Fig. 6:**
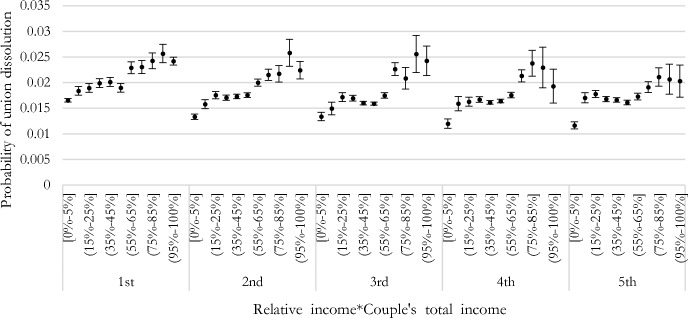
Predictive margins of union dissolution by woman’s relative income share and income quantiles, logit models (with 90% confidence intervals)

Finally, we test whether the female-breadwinner penalty is present along the total household income distribution (Fig. 6). The penalty is largest for the three lowest income quintiles and smallest for the highest income quintiles, but it is present across the whole income distribution. In addition, for lower-income quintiles, we observe a clear gradient (i.e., the higher the woman’s share of total household income, the higher the risk of union dissolution). At higher income quintiles, the gradient is lost, and the association becomes U-shaped, with equal-income couples facing a lower risk of union dissolution compared to other couple types, except for ‘sole’ male-breadwinner couples, facing the lowest risk of union dissolution of all couple types.

### The Female-Breadwinner Penalty Across Married, Cohabiting, and Registered Partnerships

**Fig. 7:**
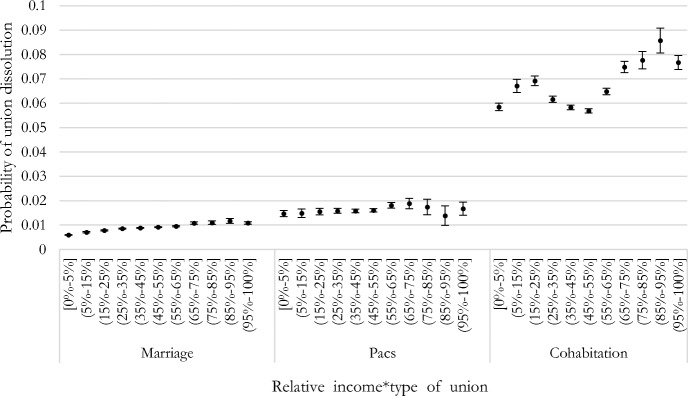
Predictive margins of union dissolution by woman’s relative income share and union type, logit models (with 90% confidence intervals)

Figure 7 shows the predicted probabilities of union dissolution, including the interaction between marital status and the woman’s share of the couple’s income. Partners in non-marital cohabitation face the highest risk of union dissolution of all couples. Registered partnerships appear to behave more similarly to married couples than to cohabiting couples, although they show a significantly higher risk of union dissolution compared to married couples. Because of different risk levels across the three couple types, we also represent the relative risks of ease of interpretation in Figure A3 in Appendix. The positive gradient between the woman’s share of total income and union dissolution is evident for married couples: the higher the female contribution to the household income, the higher the risk of divorce among married individuals. The risk of union dissolution is similar for couples in which the woman earns 45–55% and 55–65% of the couple’s total income (diff. = 0.000, *p* > 0.05), whereas it is significantly higher when her share of the couple’s incomes increases. For example, the risk of divorce is higher for couples where she earns 65–75% compared to equal-income couples (diff. = 0.001, *p* < 0.001) and to couples where she earns between 55 and 65% (diff. = 0.001, p < 0.001).

For registered partnerships, the plateau is even clearer for couples with women earning 25–55% of the couple’s income: the risk of union dissolution is the same among couples where she earns 35–45% and 25–35% (diff. = 0.000, *p* > 0.05) as well as among couples where she earns 45–55% and 35–45% (diff. = 0.000, *p* > 0.05). The female-breadwinner penalty was observed also among registered partnerships: couples with women earning between 55 and 65% of the total couples’ incomes have a higher risk of union dissolution compared to equal-income couples (diff. = 0.002, *p* < 0.05). The risk of union dissolution declines when women earn 85% and above (e.g., diff. 75–85% vs. 65–75% = − 0.01; diff. 85–95% vs. 75–85% = 0.004; diff. 95–100% vs. 75–85% = − 0.001), but such differences are not statistically significant (also, the sample size is reduced, as shown by the larger confidence intervals). Overall, the association between couples’ relative incomes and the risk of union dissolution is weaker among registered partnerships than other couple types. The association between the woman’s share of the couples’ incomes and union dissolution is completely different among cohabiting couples. Here, the similarity of incomes among partners is a stabilizing factor, and any deviation from equality of incomes is associated with an increased risk of union dissolution. For instance, equal-income couples are significantly less likely to separate compared to couples where she earns 35–45% of the couple’s income (diff = -0.001, *p* < 0.05) as well as compared to couples where she earns 55–65% (diff. = -0.008, *p* < 0.001). However, ‘sole’ male breadwinning appears to stabilize cohabiting unions; male-breadwinner couples face the lowest risk of union dissolution among all couple types.

### Female-Breadwinner Penalty by Age

**Fig. 8:**
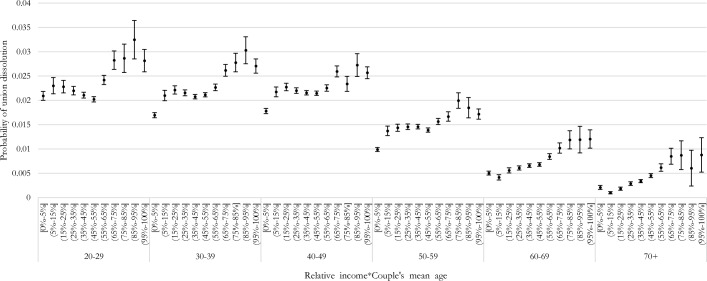
Predictive margins of union dissolution for each of the levels in the interaction of woman’s relative income share and age class, logit models (with 90% confidence intervals)

Figure 8 shows the predicted probabilities of the interaction between relative income and age (mean of both partners’ ages) on the dissolution risk. The overall risk of dissolution diminishes with age, especially after age 50. We are unable to distinguish age and cohort since our time window is too short to observe several births cohorts at the same ages. Our results hence mix cohort and age effects. Couples observed at older ages belong to older birth cohorts and are more likely to have remained in intact unions for longer (though results are robust to controlling for union duration) and hence face lower union dissolution risks. However, female-breadwinner couples face a higher risk of dissolution at all ages, including at age 70 + . Furthermore, we did not find any fading effect of the female-breadwinner penalty among younger couples, belonging to recent birth cohorts who grew-up with more egalitarian gender norms than previous generations: the female breadwinner penalty is also visible in the age group 20–29.

However, the premium for equal-income couples seems to be more pronounced for younger ages. Such a plateau does not exist for ages 70 + and becomes visible at 60 and 50 years of age. Then, the plateau becomes a couple stability premium for 40-, 30-, and 20-year- old couples. In particular, couples with employed women earning less than their partners face a higher risk of dissolution than equal-income couples. Interestingly, we also find no premium for ‘sole’ male-breadwinner couples among the youngest ages: male-breadwinner couples are equally likely to experience union dissolution as male-primary-earner couples and equal earners. This pattern is remarkably different from the one observed for other age groups, where male breadwinning appears to be a stabilizing factor.

### Control Variables and Robustness Checks

The sign of the estimated coefficients of the remaining control variables is in line with previous findings on union dissolution (see Table A1 in Appendix). Couples with at least one partner born abroad are more likely to separate. The risk of union dissolution is lower for couples with young children (under age 3) than for childless couples, but it increases with the children’s age. It is lower for home owners than for renters. It is highest in large towns of 20,000 to 200,000 inhabitants relative to rural areas, and it is higher in larger towns and the Paris area than in rural areas.

In a robustness check, we included a control for partnership duration (in years). Unfortunately, such variable is available only for married couples and couples in registered partnerships. Figure A4 presents the predicted probabilities of union dissolution obtained by interacting the partnership duration with the woman’s share of the couple’s total income. Results show that the female-breadwinner penalty persisted among couples who have been together for ten years or more. The female-breadwinner penalty is also robust to including education as a control variable in the model (Figure A5) and partners’ relative education (Figure A6).

## Discussion

Previous literature identified a female-breadwinning union dissolution penalty: couples with women out-earning their male partners are more exposed to union dissolution compared to other couples. Using a large representative sample drawn from administrative data, we tested whether such female-breadwinning union dissolution penalty also holds in France, a country where dual earning has been common and socially supported for decades. Results show that, all other things being equal, couples in which the woman’s share of the couple’s total income is higher than 55% are significantly more unstable than other couples. They are from 11 to 40% more at risk of union dissolution than equal-income couples, and the risk of union dissolution increases with the woman’s share of couple’s total income. Our study hence adds evidence that the union dissolution penalty faced by female-breadwinning couples holds also in contexts characterized by high female labor force participation and generous family policies, confirming results found by Jalovaara (2003, 2013) for Finland. It seems that the reduction in gender inequalities in the public sphere of life—e.g., men and women have similar labor market participation rates—is faster than in the private sphere, where social norms may persist for longer. One reason is that public policies have less room to take action on the private sphere.

Although we expected the female-breadwinner dissolution penalty to be less salient for unmarried couples (which are on average more egalitarian), the evidence showed that the penalty was observed for married couples, non-marital cohabitations, and registered partnerships alike. However, couple types differ substantially in terms of which income arrangement is stabilizing for the couple: a union stability premium clearly emerges when partners have roughly similar incomes among cohabiting couples; male breadwinning appears to be stabilizing among married couples, whereas the association between couples’ relative income and union dissolution is weaker among registered partnerships.

We did not find any sign of a fading effect of the female-breadwinner penalty at young ages, as suggested by previous research (Schwartz & Gonalons-Pons, 2016), but the above-mentioned stability premium for equal-income couples is especially relevant among younger ages.

The female breadwinner penalty, however, seems persistent, at least for the generations we observed, and similar to what was found by Hamplová et al. (2021) for Canada. The female-breadwinner penalty was robust also after absolute incomes and other control variables, interaction effects, and different model specifications.

Female breadwinning hides two realities: ‘sole’ female-breadwinner women with a non-working man and women as primary earners. The mechanisms behind a possible higher risk of separation for such couples compared to couples in other income arrangements might sensibly differ for these two types of couples. Both couple types deviate from ‘rigid’ gender roles, but economic precariousness and uncertainty characterize only ‘sole’ female breadwinners (Kowalewska & Vitali, 2021). We approach this issue by using different specifications. First, we isolate this group in our categorization of the woman’s share of the total couple’s income. Second, we interact the woman’s share of the couple’s income with the male employment status, distinguishing between employed, unemployed, and retired men. Third, we study the female-breadwinner penalty along the household income distribution. The penalty is largest among the lowest incomes. Results suggest that ‘sole’ female-breadwinner couples are specific because of their characteristics and different risk of dissolution: the deviation from traditional gender roles might be the main explanatory factor, whereas economic precariousness would play a secondary and additional role.

Our work also pointed out that male-breadwinner and equal-income couples are heterogeneous groups for which dissolution risk has possibly evolved. A traditional division of work (i.e., when the woman is out of the labor force or has little monetary resources) is always associated with a lower risk of dissolution. The risk of dissolution increases with the woman’s share of the couple’s total income. However, we observe an interesting “plateau” or even a trough (stability premium) for dual-earner couples where the woman’s incomes are roughly equal to the man’s incomes. This result supports the emergence of a new egalitarian equilibrium within couples who share market work equitably, particularly among the most privileged (Cherlin, 2018). Interestingly, the equal-earner stabilizing effect is indeed more salient for high-educated and high-income couples, as well as the youngest and unmarried, possibly holding more gender-egalitarian values.

Although the risk of union dissolution is always higher among female-breadwinning couples and always lower among male-breadwinner couples, equal incomes seem protective only among the youngest and the non-married couples. This result is also in line with changing norms showing that women’s employment is becoming increasingly important, and the profile of “stable couples” is changing with the diffusion of the dual-earner model.

From a methodological point of view, our results emphasize both the necessity to have high-quality data and the importance of selecting adequate comparison groups when analyzing the female-breadwinner dissolution risk. Foster and Stratton (2021) only focused on dual-earning couples where both partners have positive incomes—excluding ‘sole’ female breadwinners yields a partial picture. Further, Bertrand et al. (2015) and Schwartz and Gonalons-Pons (2016) focused only on married couples hence disregarded the experiences of non-married partnerships.

Our study has some limitations. First, our administrative data did not allow us to control for partners’ gender ideology; hence, we cannot test whether the female-breadwinner penalty disappears once gender ideology is controlled for (Gonalons-Pons & Gangl, 2021; Sayer & Bianchi, 2000). Second, our age approach was limited because the period of observation from 2010 to 2017 was not long enough to distinguish age from birth or union cohorts. Although the inclusion of union duration does not change the results for married and registered couples, we could not control for union duration for all couples to compare union cohorts. Third, although our income variables are reliable, we do not have detailed information on partners’ employment contract or working hours. Fourth, we were unable to disentangle whether the separation was initiated by the woman, e.g., to escape an unhappy union, linked to her financial autonomy, or by the man, e.g., to avoid being out-earned. To better understand such mechanisms, future studies with a qualitative approach or using surveys asking about divorce initiation are needed. However, the consistently higher dissolution rate of female-breadwinner couples in diverse circumstances is clearly an indication that the deviation from norms is difficult to accept even in countries such as France, where female employment is high and supported by family policies.

## Supplementary Information

Below is the link to the electronic supplementary material.

Supplementary file1 (DOCX 120 KB)

## Data Availability

The data that support the findings of this study are available from on a secure system (CASD) under specific conditions and so are not publicly available.
