# Supplementary material for: Are Female-Breadwinner Couples Always Less Stable? Evidence from French Administrative Data
Source: Eur J Popul. 2024 Jun 13;40(1):21. doi: 10.1007/s10680-024-09705-7 (PMC11176142; doi:10.1007/s10680-024-09705-7)
Supplement: Supplementary file 1 — Supplementary file1 (DOCX 120 KB) [file 10680_2024_9705_MOESM1_ESM.docx]

**ONLINE APPENDIX**

*Table A1. Probability of Union Dissolution, Regression Coefficients from Logistic Models*

|  | Null model | With covariates | Interaction union type | Interaction age | Interaction couple's income | Interaction man's type of income |
| --- | --- | --- | --- | --- | --- | --- |
| Relative income share (Ref: [0%-5%]) | |  |  |  |  |  |
| (5%-15%] | -0.19*** | 0.170*** | 0.178*** | 0.101* | 0.112*** | 0.238*** |
| (15%-25%] | 0.08*** | 0.249*** | 0.277*** | 0.092** | 0.145*** | 0.277*** |
| (25%-35%] | 0.23*** | 0.241*** | 0.370*** | 0.053 | 0.196*** | 0.239*** |
| (35%-45%] | 0.34*** | 0.219*** | 0.400*** | 0.008 | 0.208*** | 0.212*** |
| (45%-55%] | 0.45*** | 0.215*** | 0.438*** | -0.036 | 0.145*** | 0.195*** |
| (55%-65%] | 0.58*** | 0.320*** | 0.478*** | 0.155*** | 0.347*** | 0.292*** |
| (65%-75%] | 0.74*** | 0.461*** | 0.609*** | 0.321*** | 0.354*** | 0.445*** |
| (75%-85%] | 0.85*** | 0.484*** | 0.625*** | 0.335*** | 0.409*** | 0.432*** |
| (85%-95%] | 0.98*** | 0.561*** | 0.688*** | 0.471*** | 0.471*** | 0.551*** |
| (95%-100%] | 0.98*** | 0.458*** | 0.610*** | 0.317*** | 0.407*** | 0.481*** |
| Mean age class (Ref: 20-29) |  |  |  |  |  |  |
| 30-39 |  | -0.035*** | -0.036*** | -0.221*** | -0.035*** | -0.036*** |
| 40-49 |  | -0.017 | -0.020 | -0.170*** | -0.017 | -0.017 |
| 50-59 |  | -0.464*** | -0.467*** | -0.779*** | -0.465*** | -0.449*** |
| 60-69 |  | -1.225*** | -1.228*** | -1.467*** | -1.225*** | -1.101*** |
| 70+ |  | -1.932*** | -1.921*** | -2.359*** | -1.927*** | -1.731*** |
| Age difference (M-W) *mean* |  | 0.004*** | 0.004*** | 0.004*** | 0.003*** | 0.004*** |
| Union type (Ref: Marriage) |  |  |  |  |  |  |
| Pacs |  | 0.650*** | 0.922*** | 0.645*** | 0.649*** | 0.646*** |
| Cohabitation |  | 2.054*** | 2.366*** | 2.048*** | 2.053*** | 2.048*** |
| Age of the youngest child in the household (Ref: No children) | | |  |  |  |  |
| 0-3y |  | -0.181*** | -0.200*** | -0.196*** | -0.181*** | -0.188*** |
| 4-8y |  | 0.041*** | 0.029** | 0.031*** | 0.041*** | 0.034*** |
| 9-12y |  | 0.243*** | 0.234*** | 0.235*** | 0.243*** | 0.236*** |
| 13-18y |  | 0.501*** | 0.492*** | 0.493*** | 0.501*** | 0.494*** |
| 19y+ |  | 0.426*** | 0.417*** | 0.419*** | 0.426*** | 0.416*** |
| Unknown age |  | -0.242*** | -0.246*** | -0.243*** | -0.242*** | -0.244*** |
| Population density (Ref: Rural) |  |  |  |  |  |  |
| 2-20k |  | 0.065*** | 0.065*** | 0.065*** | 0.065*** | 0.065*** |
| 20-200k |  | 0.147*** | 0.148*** | 0.147*** | 0.147*** | 0.145*** |
| 200-2m |  | 0.111*** | 0.115*** | 0.113*** | 0.111*** | 0.110*** |
| Paris area |  | 0.053*** | 0.058*** | 0.055*** | 0.053*** | 0.051*** |
| Unknown population density |  | -0.106 | -0.114 | -0.109 | -0.105 | -0.115 |
| Both born abroad/natives |  |  |  |  |  |  |
| Mixed couples |  | 0.145*** | 0.148*** | 0.142*** | 0.145*** | 0.142*** |
| Home ownership status (Ref: Owner) |  |  |  |  |  |  |
| Social renter |  | 0.433*** | 0.434*** | 0.428*** | 0.432*** | 0.425*** |
| Private renter |  | 0.478*** | 0.480*** | 0.476*** | 0.477*** | 0.473*** |
| Quintiles of total equalized income (Ref: 1st) | |  |  |  |  |  |
| 2nd |  | -0.138*** | -0.111*** | -0.128*** | -0.227*** | -0.126*** |
| 3rd |  | -0.206*** | -0.174*** | -0.196*** | -0.223*** | -0.186*** |
| 4th |  | -0.201*** | -0.177*** | -0.195*** | -0.338*** | -0.182*** |
| 5th |  | -0.199*** | -0.173*** | -0.196*** | -0.367*** | -0.186*** |
| **Relative income share*Union type** |  |  |  |  |  |  |
| (5%-15%]*Pacs |  |  | -0.169* |  |  |  |
| (15%-25%]*Pacs |  |  | -0.223*** |  |  |  |
| (25%-35%]*Pacs |  |  | -0.294*** |  |  |  |
| (35%-45%]*Pacs |  |  | -0.328*** |  |  |  |
| (45%-55%]*Pacs |  |  | -0.349*** |  |  |  |
| (55%-65%]*Pacs |  |  | -0.266*** |  |  |  |
| (65%-75%]*Pacs |  |  | -0.356*** |  |  |  |
| (75%-85%]*Pacs |  |  | -0.452*** |  |  |  |
| (85%-95%]*Pacs |  |  | -0.748*** |  |  |  |
| (95%-100%]*Pacs |  |  | -0.479*** |  |  |  |
| (5%-15%]*Cohabitation |  |  | -0.028 |  |  |  |
| (15%-25%]*Cohabitation |  |  | -0.093*** |  |  |  |
| (25%-35%]*Cohabitation |  |  | -0.313*** |  |  |  |
| (35%-45%]*Cohabitation |  |  | -0.404*** |  |  |  |
| (45%-55%]*Cohabitation |  |  | -0.470*** |  |  |  |
| (55%-65%]*Cohabitation |  |  | -0.367*** |  |  |  |
| (65%-75%]*Cohabitation |  |  | -0.338*** |  |  |  |
| (75%-85%]*Cohabitation |  |  | -0.313*** |  |  |  |
| (85%-95%]*Cohabitation |  |  | -0.266*** |  |  |  |
| (95%-100%]*Cohabitation |  |  | -0.312*** |  |  |  |
| **Relative income share*Mean age** |  |  |  |  |  |  |
| Relative income share*Mean age class | |  |  |  |  |  |
| (5%-15%]*30-39 |  |  |  | 0.124* |  |  |
| (15%-25%]*30-39 |  |  |  | 0.189*** |  |  |
| (25%-35%]*30-39 |  |  |  | 0.199*** |  |  |
| (35%-45%]*30-39 |  |  |  | 0.205*** |  |  |
| (45%-55%]*30-39 |  |  |  | 0.268*** |  |  |
| (55%-65%]*30-39 |  |  |  | 0.152*** |  |  |
| (65%-75%]*30-39 |  |  |  | 0.138** |  |  |
| (75%-85%]*30-39 |  |  |  | 0.188** |  |  |
| (85%-95%]*30-39 |  |  |  | 0.146 |  |  |
| (95%-100%]*30-39 |  |  |  | 0.178** |  |  |
| (5%-15%]*40-49 |  |  |  | 0.110* |  |  |
| (15%-25%]*40-49 |  |  |  | 0.166*** |  |  |
| (25%-35%]*40-49 |  |  |  | 0.170*** |  |  |
| (35%-45%]*40-49 |  |  |  | 0.194*** |  |  |
| (45%-55%]*40-49 |  |  |  | 0.233*** |  |  |
| (55%-65%]*40-49 |  |  |  | 0.094** |  |  |
| (65%-75%]*40-49 |  |  |  | 0.078 |  |  |
| (75%-85%]*40-49 |  |  |  | -0.047 |  |  |
| (85%-95%]*40-49 |  |  |  | -0.020 |  |  |
| (95%-100%]*40-49 |  |  |  | 0.071 |  |  |
| (5%-15%]*50-59 |  |  |  | 0.237*** |  |  |
| (15%-25%]*50-59 |  |  |  | 0.293*** |  |  |
| (25%-35%]*50-59 |  |  |  | 0.347*** |  |  |
| (35%-45%]*50-59 |  |  |  | 0.394*** |  |  |
| (45%-55%]*50-59 |  |  |  | 0.386*** |  |  |
| (55%-65%]*50-59 |  |  |  | 0.320*** |  |  |
| (65%-75%]*50-59 |  |  |  | 0.220*** |  |  |
| (75%-85%]*50-59 |  |  |  | 0.394*** |  |  |
| (85%-95%]*50-59 |  |  |  | 0.177 |  |  |
| (95%-100%]*50-59 |  |  |  | 0.255*** |  |  |
| (5%-15%]*60-69 |  |  |  | -0.295*** |  |  |
| (15%-25%]*60-69 |  |  |  | 0.014 |  |  |
| (25%-35%]*60-69 |  |  |  | 0.140* |  |  |
| (35%-45%]*60-69 |  |  |  | 0.263*** |  |  |
| (45%-55%]*60-69 |  |  |  | 0.342*** |  |  |
| (55%-65%]*60-69 |  |  |  | 0.371*** |  |  |
| (65%-75%]*60-69 |  |  |  | 0.398*** |  |  |
| (75%-85%]*60-69 |  |  |  | 0.541*** |  |  |
| (85%-95%]*60-69 |  |  |  | 0.409** |  |  |
| (95%-100%]*60-69 |  |  |  | 0.574*** |  |  |
| (5%-15%]*70+ |  |  |  | -0.838*** |  |  |
| (15%-25%]*70+ |  |  |  | -0.223 |  |  |
| (25%-35%]*70+ |  |  |  | 0.272* |  |  |
| (35%-45%]*70+ |  |  |  | 0.483*** |  |  |
| (45%-55%]*70+ |  |  |  | 0.818*** |  |  |
| (55%-65%]*70+ |  |  |  | 0.945*** |  |  |
| (65%-75%]*70+ |  |  |  | 1.105*** |  |  |
| (75%-85%]*70+ |  |  |  | 1.115*** |  |  |
| (85%-95%]*70+ |  |  |  | 0.608 |  |  |
| (95%-100%]*70+ |  |  |  | 1.141*** |  |  |
| **Relative income share*Quintiles of total equalized income** | | |  |  |  |  |
| (5%-15%]*2nd |  |  |  |  | 0.065 |  |
| (15%-25%]*2nd |  |  |  |  | 0.145*** |  |
| (25%-35%]*2nd |  |  |  |  | 0.062 |  |
| (35%-45%]*2nd |  |  |  |  | 0.065 |  |
| (45%-55%]*2nd |  |  |  |  | 0.144*** |  |
| (55%-65%]*2nd |  |  |  |  | 0.081* |  |
| (65%-75%]*2nd |  |  |  |  | 0.152*** |  |
| (75%-85%]*2nd |  |  |  |  | 0.107 |  |
| (85%-95%]*2nd |  |  |  |  | 0.233*** |  |
| (95%-100%]*2nd |  |  |  |  | 0.144** |  |
| (5%-15%]*3rd |  |  |  |  | 0.003 |  |
| (15%-25%]*3rd |  |  |  |  | 0.118** |  |
| (25%-35%]*3rd |  |  |  |  | 0.052 |  |
| (35%-45%]*3rd |  |  |  |  | -0.021 |  |
| (45%-55%]*3rd |  |  |  |  | 0.035 |  |
| (55%-65%]*3rd |  |  |  |  | -0.066 |  |
| (65%-75%]*3rd |  |  |  |  | 0.203*** |  |
| (75%-85%]*3rd |  |  |  |  | 0.060 |  |
| (85%-95%]*3rd |  |  |  |  | 0.219* |  |
| (95%-100%]*3rd |  |  |  |  | 0.225** |  |
| (5%-15%]*4th |  |  |  |  | 0.184** |  |
| (15%-25%]*4th |  |  |  |  | 0.176*** |  |
| (25%-35%]*4th |  |  |  |  | 0.151** |  |
| (35%-45%]*4th |  |  |  |  | 0.103* |  |
| (45%-55%]*4th |  |  |  |  | 0.183*** |  |
| (55%-65%]*4th |  |  |  |  | 0.055 |  |
| (65%-75%]*4th |  |  |  |  | 0.253*** |  |
| (75%-85%]*4th |  |  |  |  | 0.316*** |  |
| (85%-95%]*4th |  |  |  |  | 0.216 |  |
| (95%-100%]*4th |  |  |  |  | 0.095 |  |
| (5%-15%]*5th |  |  |  |  | 0.286*** |  |
| (15%-25%]*5th |  |  |  |  | 0.299*** |  |
| (25%-35%]*5th |  |  |  |  | 0.186*** |  |
| (35%-45%]*5th |  |  |  |  | 0.163*** |  |
| (45%-55%]*5th |  |  |  |  | 0.194*** |  |
| (55%-65%]*5th |  |  |  |  | 0.066 |  |
| (65%-75%]*5th |  |  |  |  | 0.166** |  |
| (75%-85%]*5th |  |  |  |  | 0.216*** |  |
| (85%-95%]*5th |  |  |  |  | 0.132 |  |
| (95%-100%]*5th |  |  |  |  | 0.177 |  |
| **Man's type of income (Ref: wage+self empl.)** | |  |  |  |  |  |
| unemployed / no income |  |  |  |  |  | 0.214*** |
| retired |  |  |  |  |  | -0.209*** |
| Relative income share*Man's type of income | |  |  |  |  |  |
| (5%-15%]*unemployed / no income |  |  |  |  |  | -0.130 |
| (15%-25%]*unemployed / no income |  |  |  |  |  | -0.070 |
| (25%-35%]*unemployed / no income |  |  |  |  |  | 0.048 |
| (35%-45%]*unemployed / no income |  |  |  |  |  | -0.068 |
| (45%-55%]*unemployed / no income |  |  |  |  |  | 0.026 |
| (55%-65%]*unemployed / no income |  |  |  |  |  | -0.099** |
| (65%-75%]*unemployed / no income |  |  |  |  |  | -0.184*** |
| (75%-85%]*unemployed / no income |  |  |  |  |  | -0.051 |
| (85%-95%]*unemployed / no income |  |  |  |  |  | -0.151* |
| (95%-100%]*unemployed / no income |  |  |  |  |  | -0.235*** |
| (5%-15%]*retired |  |  |  |  |  | -0.633*** |
| (15%-25%]*retired |  |  |  |  |  | -0.278*** |
| (25%-35%]*retired |  |  |  |  |  | 0.003 |
| (35%-45%]*retired |  |  |  |  |  | 0.098** |
| (45%-55%]*retired |  |  |  |  |  | 0.178*** |
| (55%-65%]*retired |  |  |  |  |  | 0.248*** |
| (65%-75%]*retired |  |  |  |  |  | 0.217*** |
| (75%-85%]*retired |  |  |  |  |  | 0.288*** |
| (85%-95%]*retired |  |  |  |  |  | 0.180* |
| (95%-100%]*retired |  |  |  |  |  | 0.309* |
| Constant | -4.375*** | -4.804*** | -4.950*** | -4.620*** | -4.767*** | -4.807*** |
| Observations | 5,536,503 | 5,536,503 | 5,536,503 | 5,536,503 | 5,536,503 | 5,536,503 |
| *** p<0.01, ** p<0.05, * p<0.1 |  |  |  |  |  |  |

Figure A2 Risk of union dissolution by woman’s share of couples’ incomes

Note: Dotted line represents the yearly dissolution risk for the 101 levels of income share (from 0 to 100) whereas the solid line indicates the moving average of 5%.

**Figure A3: Risk-ratio of union dissolution by woman’s relative income share and union type, logit models** (with 90% Confidence Intervals)

Note: the risk ratio compares the dissolution risk, taking the share (45-55] as reference category.

For example, married couple whose woman earn between 65 and 75 are 22% more likely to separate than married couple with partners having similar incomes.

Figure A4: Predictive margins of union dissolution by woman’s relative income share and union duration (subsample of married and registered partnerships only), logit models (with 90% Confidence Intervals)

Figure A5: Predictive margins of union dissolution by woman’s relative income share and woman’s educational level, logit models (with 90% Confidence Intervals)

Figure A6: Predictive margins of union dissolution by woman’s relative income share and partners’ relative education, logit models (with 90% Confidence Intervals)
